# Transcriptomic Profiling Reveals Complex Molecular Regulation in Cotton Genic Male Sterile Mutant Yu98-8A

**DOI:** 10.1371/journal.pone.0133425

**Published:** 2015-09-18

**Authors:** Weiping Fang, Fu'an Zhao, Yao Sun, Deyi Xie, Li Sun, Zhenzhen Xu, Wei Zhu, Lirong Yang, Yuanming Zhao, Shuping Lv, Zhongjie Tang, Lihong Nie, Wu Li, Jianan Hou, Zhengzheng Duan, Yuebo Yu, Xiaojie Yang

**Affiliations:** 1 Economic Crop Research Institute, Henan Academy of Agriculture Sciences, Zhengzhou, Henan province, 450002, R.P. China; 2 Department of Biological Sciences, Texas Tech University, Lubbock, Texas, United States of America; 3 State Key Laboratory of Cotton Biology, Cotton Research Institute, Chinese Academy of Agriculture Sciences, Anyang, Henan province, 455000, R.P. China; 4 Agronomy College, Henan Agricultural University, Zhengzhou, Henan province, 450002, R.P. China; 5 Plant Protection Research Institute, Henan Academy of Agriculture Sciences, Zhengzhou, Henan province, 450002, R.P. China; USDA-ARS-SRRC, UNITED STATES

## Abstract

Although cotton genic male sterility (GMS) plays an important role in the utilization of hybrid vigor, its precise molecular mechanism remains unclear. To characterize the molecular events of pollen abortion, transcriptome analysis, combined with histological observations, was conducted in the cotton GMS line, Yu98-8A. A total of 2,412 genes were identified as significant differentially expressed genes (DEGs) before and during the critical pollen abortion stages. Bioinformatics and biochemical analysis showed that the DEGs mainly associated with sugars and starch metabolism, oxidative phosphorylation, and plant endogenous hormones play a critical and complicated role in pollen abortion. These findings extend a better understanding of the molecular events involved in the regulation of pollen abortion in genic male sterile cotton, which may provide a foundation for further research studies on cotton heterosis breeding.

## Introduction

Cotton, an economically important crop cultivated in more than 80 countries around the world, is the most important natural source of fiber for textiles and relatively high-quality protein and oil [1]. Cotton heterosis has the potential of increasing yield from 10% to 20%, and has substantially remained as one of the significant developments in cotton breeding programs. Therefore, morphological [2,3], biochemical [4,5], and molecular aspects of cotton male abortion mechanism continues to be a highly interesting research area [6–8].

Genic male sterility (GMS), which is one of the two pollination control systems in plants, has been widely exploited in the utilization of hybrid vigor based on its unique advantages. For example, the recessive sterile gene itself determines most breeding lines can be served as its restorers, therefore, it is easy to combine elite lines to produce hybrids that show high heterosis. In addition, genic male sterile genes are relatively easy to transfer to any desired genetic background because this type of male sterility pertains only to nuclear genes [9]. Although a two line-system has recently become an important breeding strategy, the mechanism of sterility of pollen in cotton has yet to be extensively investigated. One novel cotton male sterile mutant, Yu98-8A, the sterility of which is controlled by a pair of recessive genes, displays a strong heterosis in various combinations [10,11]. In addition, because of the same genetic background, Yu98-8A could be used as an ideal genetic material in investigating the molecular regulatory networks, and mechanisms underlying the pollen abortion process in male sterility in cotton.

RNA-seq, a next-generation sequencing technology, has made a breakthrough in the life sciences by offering a highly accurate quantification of expression analysis with a low background signal [12]. In recent years, RNA-seq has been used to study the mechanism underlying male sterility in various crops such as rape and chili pepper [13,14]. In the case of genic male sterile of cotton, one research has identified several key genes involved in various aspects of anther development that followed a gene expression pattern in the genic male sterile mutant, indicating that diverse gene regulation pathways are involved in the GMS mutant anther development [15]. In the present study, the next-generation sequencing approach, combined with was applied to create a more complete survey of transcriptome dynamics in cotton GMS in using mutant Yu98-8A. The data generated in this study may serve as a foundational resource for future studies addressing fundamental molecular and developmental mechanisms from pollen mother cell formation to meiotic stages during cotton male sterile development.

## Results and Discussion

### Morphology and histology of cotton male genic sterile (MS) and fertile (MF) buds

**Fig 1:**
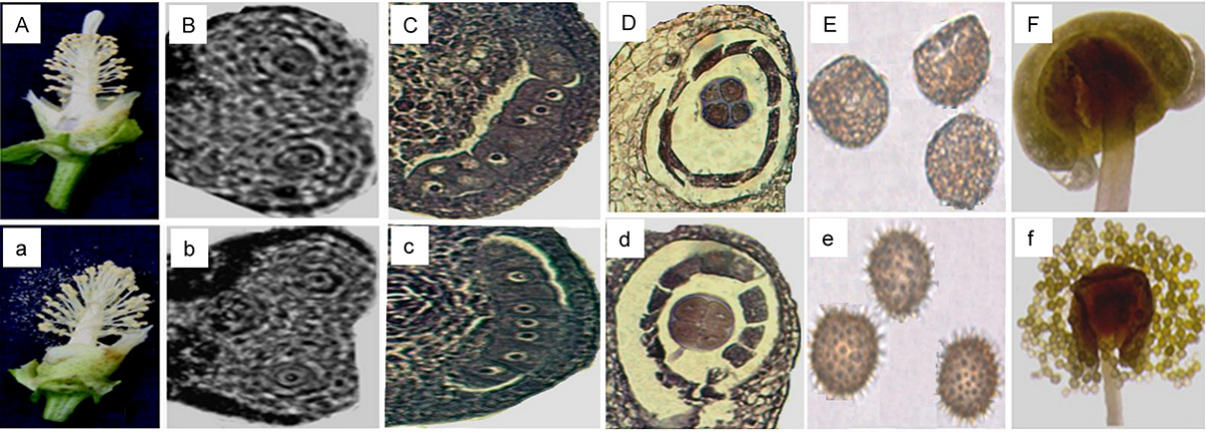
Phenotypic and microscopic observation during pollen development between genic male sterile mutant (MS) and normal wild-type (MF) flowers. A and a represent the exposed stigma and normal phenotype of GMS mutant and wild-type flowers, respectively. B and b, and C and c represent the sporogenous cell (MS: ≤1.8 mm, MF: ≤2.0 mm) and pollen mother cell formation stages (MS: 1.8–2.5 mm, MF: 2.0–2.8 mm), both of which showed highly similar phenotypes between the GMS mutant and wild-type buds. D, E, F and d, e, f represent microspore tetrads during meiosis (MS: 2.5–3.0 mm, MF: 2.8–3.3 mm), pollen grains and anthers (MS: 3.0–5.0 mm, MF: 3.3–5.5 mm) of GMS mutant and wild-type buds, respectively.

On the day of anthesis (DPA), cotton genic male sterile mutant (MS) Yu98-8A showed abnormal floral phenotypes with longer and exposed stigma (Fig 1A), whereas the corresponding wild-type (MF) showed normal floral phenotypes (Fig 1a). Microscopic observation showed that, from sporogenous cell (MS: ≤ 1.8 mm, MF: ≤ 2.0 mm) and pollen mother cell formation (MS: 1.8–2.5 mm, MF: 2.0–2.8 mm) stages, no significant phenotypic differences between MS and MF buds were observed (Fig 1B, 1b, 1C and 1c). However, during meiosis stages, especially at the point of tetrad formation (MS: 2.5–3.0 mm, MF: 2.8–3.3 mm), compared to the normal tetrad of wild-type and pollen (Fig 1d and 1e), Yu98-8A showed a shriveled tetrad and no spinescent protuberances on the pollen wall (Fig 1D and 1E). In addition, abnormal and fragmented microspores were observed, subsequently resulted in the formation of anther sacs without pollen grains finally (Fig 1E and 1F). These results showed the shriveled tetrad was the first significant microscopic phenotype that developed, indicating that the pollen mother cell formation phase and the following meiotic cycle are vital periods of microspores abortion in MS Yu98-8A.

### Transcriptome sequencing, assembly, and annotation of four libraries

To identify the differences in gene expression patterns between the male sterile MS and corresponding MF buds, the buds were harvested during the pollen mother cell formation stage (WT: F-1, GMS: S-1) and the meiotic cycle (WT: F-2, GMS: S-2) to construct four libraries based on the microscopic features. As a result, 11,241,020, 12,247,449, 11,922,046, and 11, 942,988 reads with a Q30-value of > 85% were generated from F-1, S-1, F-2 and S-2 libraries by RNA-seq, respectively. The alignment between all the reads and *Gossypium*. *hirsutum* unigenes (http://occams.dfci.harvard.edu/pub/bio/tgi/data/Gossypium) showed that, in general, the mapping ratio were higher than 76% (S1 Table). In addition, 98,305 transcripts and 44,804 unigenes were achieved by Trinity *de novo* assembly using the paired-end splicing mode [16], and the N50 of which were 1,609 and 1,456, respectively. Furthermore, an online analysis tool (http://emboss.sourceforge.net/apps/cvs/emboss/apps/getorf.html) was applied to identify the open reading frames (ORFs) of these assembled unigenes from the four libraries based on the strategy that both forward and backward ORFs from the corresponding first three bases were predicted. Finally, the longest ORFs were observed in 44,804 unigenes, which indicated that the integrity of the *de novo* assembly was reliable (S2 Table, S1 File).

To estimate whether the sequencing depth provided sufficient transcriptome coverage, sequencing saturation analysis of each library was performed. When the sequencing counts reached about 2 million tags or higher, the number of detected genes sustained saturation, which indicated these data were all sufficient for quantitative analysis of gene expression (S1 Fig). In addition, sequence alignment between each library and the assembly of total unigenes using Bowtie showed that the mapping ratios of each library were about 78% (S1 Table) [17], which also indicated that both sequencing quality and alignment were normal.

To further assign putative functions, a set of sequential BLAST searches of all the assembled 44,804 unigenes against with sequences in the National Center for Biotechnology Information (NCBI) non-redundant proteins and nucleotides, Swiss-Prot proteins, the Gene Ontology, the Cluster of Orthologous Groups, and Kyoto Encyclopedia of Genes and Genomes databases was performed [18–20], which indicated that a total of 34,220 (76.38%) unigenes were successfully annotated (Table 1).

**Table 1:** Annotation of all the assembled unigenes from four libraries.

Annotation database	Annotated number	300 ≤ length < 1,000	Length ≥ 1,000
COG	10,590	3,697	5,504
GO	26,121	10,902	10,560
KEGG	7,996	3,344	3,111
Swiss-prot	22,605	9,481	9,172
nr	34,153	15,123	12,543
All annotated	34,220	15,145	12,545

### Global analysis of differentially expressed genes between MS and MF buds

**Fig 2:**
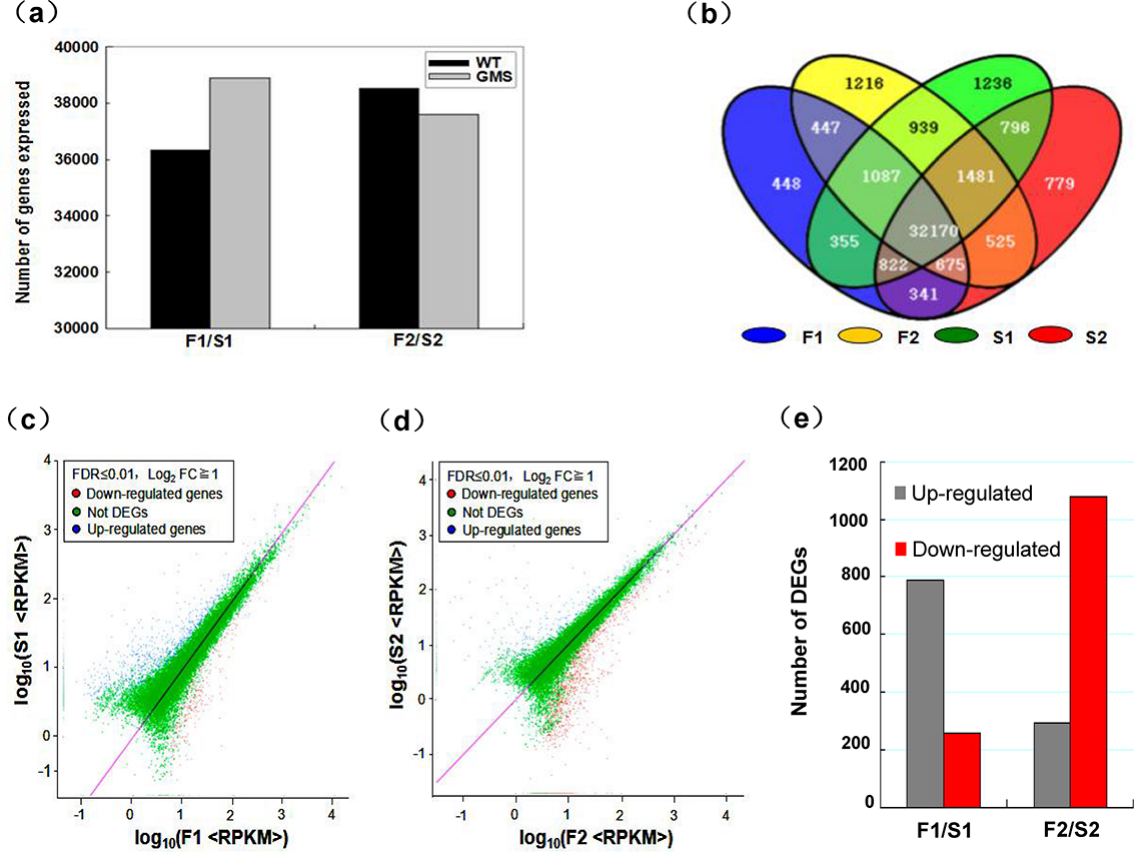
Transcriptome and differentially expressed gene (DEG) analysis of the wild-type and genic male sterile mutant buds. (a) shows the transcriptome sizes at two periods, from meiosis to the formation of tetrads, and from the release of microspores to the formation of spinescent protuberances in wild-type and genic MS mutant anthers, respectively. (b) shows the mutual overlaps of the two bud development stages in wild-type and genic male sterile mutant buds, and that of the two stages of each of the two buds. (c) and (d) show the expressed genes at the two stages in wild-type and genic MS mutant anthers. (e) shows the number of differentially expressed genes at the two stages in wild-type and genic MS mutant anthers.

Based on deep sequencing of the four cDNA libraries, 36,345 and 38,886 genes were detected during the pollen mother cell formation stage of MF and MS anthers, respectively, and 38,540 and 37,589 genes were also detected from the meiotic cycle, respectively (Fig 2a). Each stage of WT and mutant buds expressed more than 30,000 genes, which was higher than that observed in cotton [15], but similar to that of anthers of other plants [21]. Strikingly, although the MS buds were defective, the transcriptome showed a highly dynamic pattern. The buds during pollen mother cell formation expressed more than 2,541 genes compared to that observed in the WT buds, which indicated that MS pollen development might have some unique gene regulatory processes and metabolic pathways other than that observed in MF pollen development. Spatial analysis was also performed on differentially expressed genes to ascertain the degree of overlap between the two different periods during pollen development. From a total of 43,317 expressed genes, 4.87% (2,111/43,317) were MF-specifically expressed, and 6.49% (2,813/43,317) were MS-specifically expressed during pollen development (Fig 2b).

Differentially expressed genes in mutant MS and normal MF buds were identified according to the method of reads per kilobase per million mapped reads (RPKM), which considered the impact of sequencing depth and gene length on read counts. On the basis of the applied criteria [q-value < 0.01 and log_2_ (fold-change) > 1], a total of 2,412 genes were identified as significantly differentially expressed genes between WT and MS buds. Among those genes, compared with the expressed genes of WT buds, 786 (73%, 1,076) were upregulated and 290 (27%, 1,076) were downregulated in MS buds at the pollen mother formation stage, and 254 (19%, 1,336) were upregulated and 1,082 (81%, 1,336) were downregulated during meiosis (Fig 2c, 2d, 2e, and S2 Fig).

### Validation of DGE tag data by qRT-PCR

**Fig 3:**
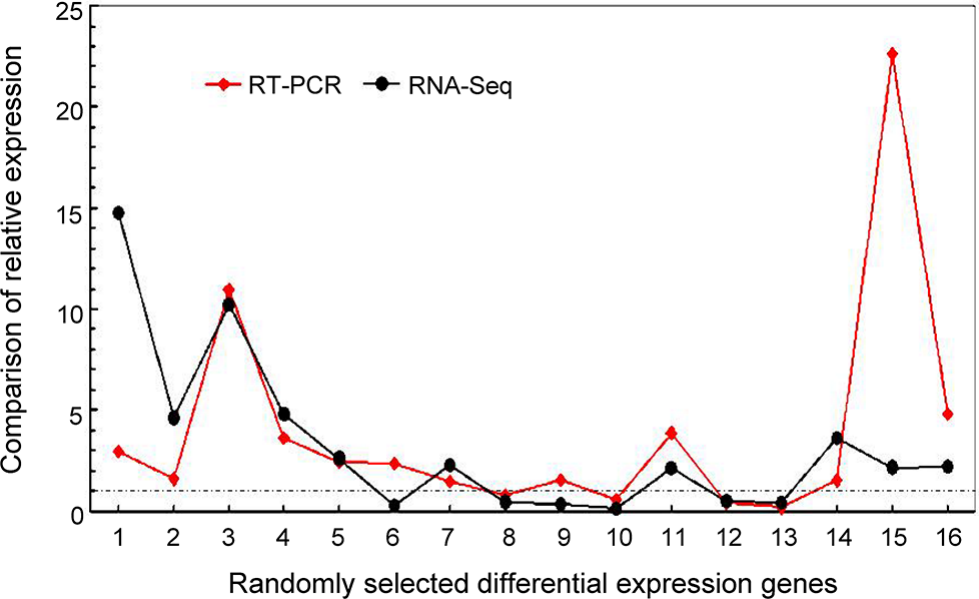
Comparison of RNA-seq data with quantitative RT-PCR results. The relative expression levels during the pollen mother cell formation stage between the normal development buds and the MS buds between of randomly selected genes identified by both RNA-seq and quantitative RT-PCR, respectively. The numbers 1–16 represents the randomly selected DEGs identified by RNA-seq, which represent the calcium-binding protein (ID: Cotton_D_gene_10025846), pollen-specific protein (ID: Cotton_D_gene_10008857), GA dioxygenase (ID: Cotton_D_gene_10009218), ABA hydroxylase (ID: Cotton_D_gene_10024447), cyclin kinase inhibitor (ID: Cotton_D_gene_10012996), ET transcription factor (ID: Cotton_D_gene_10032583), GA dioxygenase (ID: Cotton_D_gene_10023237), ABA hydroxylase (ID: Cotton_D_gene_10016475), GA transcript protein (ID: Cotton_D_gene_10025900), Auxin responsive SAUR protein (ID: Cotton_D_gene_10040607), ET transcription factor (ID: Cotton_D_gene_10025875), Gibberellin-regulated (ID: Cotton_D_gene_10024873), pollen-specific protein (ID: Cotton_D_gene_10009412), pollen allergen protein (ID: Cotton_D_gene_10006155), asparagine synthetase (ID: Cotton_D_gene_10006550), and IAA-induced protein (ID: Cotton_D_gene_10027147), respectively. The primer sequences used in quantitative RT-PCR for each gene are listed in S3 Table.

To validate the expression profiles of assembled sequences and obtained by RNA-seq, real-time RT-PCR was performed on 16 differentially expressed genes, including calcium-binding protein (ID: Cotton_D_gene_10025846), pollen-specific protein (ID: Cotton_D_gene_10008857), gibberellin 2-β-dioxygenase 1 (ID: Cotton_D_gene_10009218), cyclin-dependent protein kinase inhibitor (ID: Cotton_D_gene_10012996), ethylene-responsive transcription factor (ID: Cotton_D_gene_10032583), asparagine synthetase (ID: Cotton_D_gene_10006550), and indole-3-acetic acid-induced protein (ID: Cotton_D_gene_10027147), and so on (S3 Table). Although quantitative differences existed between the quantitative RT-PCR and the RNA-seq data were observed, the overall tendencies of most selected DEGs were the same. For the 16 detected genes, the upregulation patterns obtained in real-time RT-PCR expression analysis were all in agreement with the RNA-seq data, except for unigene (ID: Cotton_D_gene_10032583), which encodes for a ethylene-responsive transcription factor (6), and unigene (ID: Cotton_D_gene_10025900) which encodes a GA-stimulated transcript-like protein (9) (Fig 3). Taken together, although 12.5% (2/16) of the genes showed the opposing patterns in the two analytic methods probably due to varying buds collection years, we believed the RT-PCR results confirmed the reliability of our transcriptome analysis by RNA-seq.

### Genes associated with male sterility in GMS mutant buds

**Fig 4:**
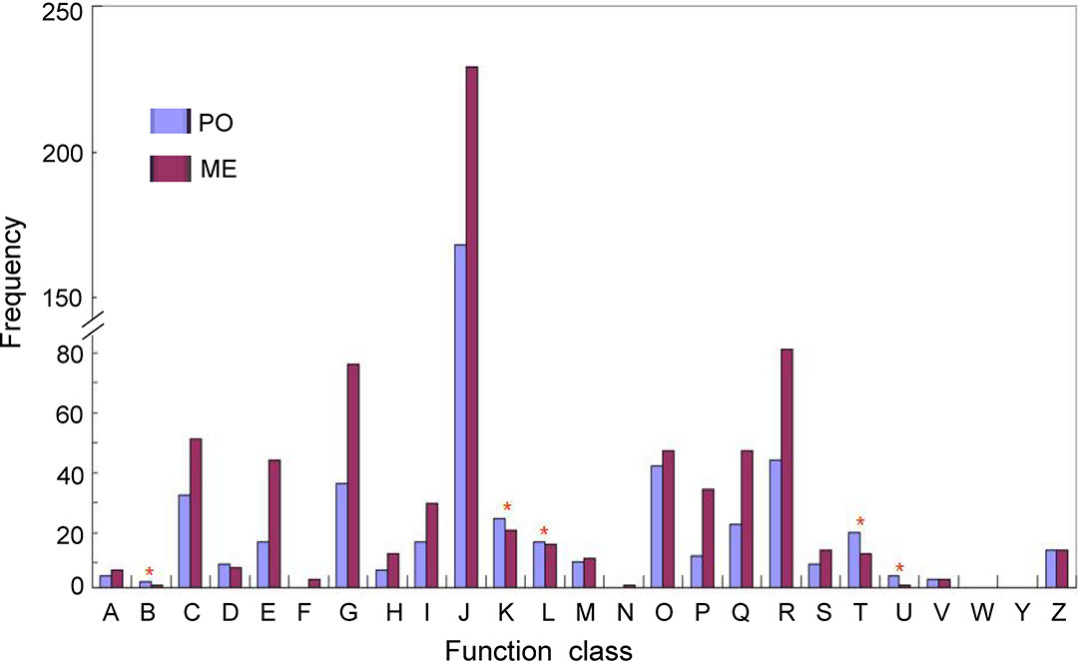
COG function classification of differentially expressed consensus sequence during pollen mother cell formation (PO) and meiosis (ME) stages. A-V and Z represent RNA processing and modification (A), chromatin structure and dynamics (B), energy production and conversation (C), cell cycle control, cell division, chromosome partitioning (D), amino acid transport and metabolism (E), nucleotide transport and metabolism (F), carbohydrate transport and metabolism (G), coenzyme transport and metabolism (H), lipid transport and metabolism (I), translation, ribosomal structure and biogenesis (J), transcription (K), replication, recombination and repair (L), cell wall/membrane/envelope biogenesis (M), cell motility (N), post-translational modification, protein turnover, and chaperones (O), inorganic transport, and metabolism (P), secondary metabolite biosynthesis, transport and catabolism (Q), general function prediction only (R), function unknown (S), signal transduction mechanism (T), intracellular trafficking, secretion, and vesicular transport (U), and cytoskeleton (Z).

To further analyze the annotated DEGs associated with GMS in MS mutant buds, COG analysis was performed for to identify the major functions of genes involved in genic MS line Yu 98-8A at the pollen mother cell formation and meiosis stages, respectively. At the meiosis stage, a total of 757 annotated DEGs were divided into 23 specific categories, and among these 23 COG categories, translation, ribosomal structure, and biogenesis catagory (229, 30.3%) showed the highest number, followed by general function prediction only (82, 10.8%), carbohydrate transport and metabolism (77, 10.2%), energy production and conversation (51, 6.7%), posttranslational modification, protein turnover, and chaperones (47, 6.2%), and secondary metabolites biosynthesis, transport and catabolism (47, 6.2%). Compared to the distribution of DEGs at the meiosis stage, a total of 503 annotated DEGs were divided into 21 specific categories during pollen mother formation stage. Although nucleotide transport and metabolism, and cell motility categories showed no annotated DEGs at this stage, all the categories of chromatin structure and dynamics (B), cell cycle control, cell division, chromosome partitioning (D), transcription (K), replication, recombination and repair (L), signal transduction mechanism (T), and intracellular trafficking, secretion, and vesicular transport (U) showed more DEGs than that observed during the meiosis stage (Fig 4).

To further and comprehensively demonstrate and identify the functions of DEGs in specific biological pathways, KEGG enrichment analysis was performed. In total, among the 5,736 unigenes that were assigned to 82 different specific metabolic pathways, 289 DEGs were assigned to 72 pathways at the pollen mother cell formation stage, and 408 DEGs to 71 pathways at the meiosis stage (Table 2).

**Table 2:** KEGG pathways of DEGs at both pollen mother cell formation and meiosis stages.

Enriched KEGG pathways		DEGs of all genes		P_value
No.	Enriched pathway	PO stage	ME stage	
1	Ribosome (Ko03010)	146^a^/683^b^	197/683	9.1794e^-63^
2	Oxidative phosphorylation (Ko00190)	16/272	18/272	2.9516e^-01^
3	Protein processing in endoplasmic reticulum (Ko04141)	14/339	20/339	8.1910e^-01^
4	Glycolysis/gluconeogenesis (Ko00010)	13/247	21/247	4.7706e^-01^
5	Carbon fixation in photosynthetic organisms (Ko00710)	10/194	18/194	5.1774e^-01^
6	Endocytosis (Ko04144)	9/136	5/136	2.4611e^-01^
7	Nitrogen metabolism (Ko00910)	8/63	12/63	1.3057e^-02^
8	Spliceosome (Ko03040)	8/220	9/220	8.7360e^-01^
9	RNA transport (Ko03013)	7/249	7/249	9.7204e^-01^
10	Starch and sucrose metabolism (Ko00500)	6/188	13/188	9.1917e^-01^
11	Steroid biosynthesis (Ko00100)	6/46	2/46	2.6640e^-02^
12	Valine, leucine, and isoleucine degradation (Ko00280)	6/78	5/78	1.9849e^-01^
13	Pyruvate metabolism (Ko00620)	6/179	9/179	8.9533e^-01^
14	Terpenoid backbone biosynthesis (Ko00900)	6/67	4/67	1.1987e^-01^
15	Glutathione metabolism (Ko00480)	6/102	6/102	4.0917e^-01^
16	Arginine and proline metabolism (Ko00330)	6/116	8/116	5.3400e^-01^
17	Glyoxylate and dicarboxylate metabolism (Ko00630)	5/90	11/90	4.7778e^-01^
18	Phagosome (Ko04145)	5/153	7/153	8.9189e^-01^
19	Galactose metabolism (Ko00052)	5/74	10/74	3.1643e^-01^
20	Amino sugar and nucleotide sugar metabolism (Ko00520)	5/157	4/157	9.0421e^-01^
21	Alanine, aspartate, and glutamate metabolism (Ko00250)	5/75	11/75	3.2650e^-01^
22	Pentose phosphate pathway (Ko00030)	4/103	9/103	7.7045e^-01^
23	Pentose and glucuronate interconversions (Ko00040)	4/93	5/93	6.9711e^-01^
24	Phenylpropanoid biosynthesis (Ko00940)	4/98	4/98	7.3568e^-01^
25	Butanoate metabolism (Ko00650)	4/38	1/38	1.2222e^-01^
26	Citrate cycle (Ko00020)	4/120	7/120	8.6239e^-01^
27	Plant-pathogen interaction (Ko04626)	4/191	7/191	9.8899e^-01^
28	Fructose and mannose metabolism (Ko00051)	4/83	10/83	6.0850e^-01^
29	RNA degradation (Ko03018)	4/153	4/153	9.5459e^-01^
30	Purine metabolism (Ko00230)	4/181	1/181	9.8382e^-01^
31	Tryptophan metabolism (Ko00380)	4/61	5/61	3.6961e^-01^
32	mRNA surveillance pathway (Ko03015)	3/152	3/152	9.8496e^-01^
33	Carotenoid biosynthesis (Ko00906)	3/49	3/49	4.5175e^-01^
34	Phosphatidylinositol signaling system (Ko04070)	3/80	1/80	7.7592e^-01^
35	Phenylalanine metabolism (Ko00360)	3/90	3/90	8.3960e^-01^
36	Plant hormone signal transduction (Ko04075)	3/316	4/316	9.9999e^-01^
37	Cysteine and methionine metabolism (Ko00270)	3/132	5/132	9.6628e^-01^
38	Peroxisome (Ko04146)	3/117	7/117	9.3971e^-01^
39	Synthesis and degradation of ketone bodies (Ko00072)	3/10	0/0	1.1660e^-02^
40	Valine, leucine, and isoleucine biosynthesis (Ko00290)	3/55	2/55	5.2928e^-01^
41	Fatty acid metabolism (Ko00071)	2/72	2/72	8.8500e^-01^
42	Propanoate metabolism (Ko00640)	2/71	3/71	8.8017e^-01^
43	Glycerolipid metabolism (Ko00561)	2/72	7/72	8.8500e^-01^
44	Lysine degradation (Ko00310)	2/35	2/35	5.3284e^-01^
45	Biosynthesis of unsaturated fatty acids (Ko01040)	2/61	7/61	8.2064e^-01^
46	Ascorbate and aldarate metabolism (Ko00053)	2/67	2/67	8.5895e^-01^
47	Stilbenoid, diarylheptanoid, and gingerol biosynthesis (Ko00945)	2/18	0/0	2.2898e^-01^
48	Flavonoid biosynthesis (Ko00941)	2/33	1/33	5.0104e^-01^
49	Inositol phosphate metabolism (Ko00562)	2/90	1/90	9.4623e^-01^
50	Pantothenate and CoA biosynthesis (Ko00770)	2/38	1/38	5.7789e^-01^
51	Photosynthesis (Ko00195)	2/158	21/158	9.9757e^-01^
52	Selenocompound metabolism (Ko00450)	2/25	0/0	3.6133e^-01^
53	Ubiquitin mediated proteolysis (Ko04120)	2/191	3/191	9.9950e^-01^
54	Porphyrin and chlorophyll metabolism (Ko00860)	1/63	2/63	9.6219e^-01^
55	Pyrimidine metabolism (Ko00240)	1/142	2/142	9.9941e^-01^
56	Diterpenoid biosynthesis (Ko00904)	1/18	0/0	6.0622e^-01^
57	β-Alanine metabolism (Ko00410)	1/46	3/46	9.0816e^-01^
58	Tyrosine metabolism (Ko00350)	1/44	1/44	8.9807e^-01^
59	Arachidonic acid metabolism (Ko00590)	1/16	1/16	5.6319e^-01^
60	Base excision repair (Ko03410)	1/41	0/0	8.8083e^-01^
61	Circadian rhythm-mammal (Ko047 10)	1/23	2/23	6.9620e^-01^
62	Glycosaminoglycan degradation (Ko00531)	1/17	1/17	5.8526e^-01^
63	Natural killer cell-mediated cytotoxicity (Ko04650)	1/26	0/0	7.4002e^-01^
64	Glycerophospholipid metabolism (Ko00564)	1/76	2/76	9.7089e^-01^
65	Mismatch repair (Ko03430)	1/41	0/0	8.8083e^-01^
66	Tropane, piperidine, and pyridine alkaloid biosynthesis (Ko00960)	1/17	0/0	5.8526e^-01^
67	Glycine, serine, and threonine metabolism (Ko00260)	1/68	3/68	9.8085e^-01^
68	Nucleotide excision repair (Ko03420)	1/56	0/0	9.4549e^-01^
69	DNA replication (Ko03030)	1/58	0/0	9.5090e^-01^
70	Limonene and pinene degradation (Ko00903)	1/26	2/26	7.4002e^-01^
71	Histidine metabolism (Ko00340)	1/33	2/33	8.1930e^-01^
72	Homologous recombination (Ko03440)	1/49	0/0	9.2146e^-01^
73	Photosynthesis-antenna proteins (ko00196)	0/0	11/54	1.2090e^-03^
74	Fatty acid biosynthesis (ko00061)	0/0	6/60	2.5265e^-01^
75	Glycosphingolipid biosynthesis-globo series (ko00603)	0/0	3/20	1.6627e^-01^
76	Sphingolipid metabolism (ko00600)	0/0	3/26	2.8084e^-01^
77	N-Glycan biosynthesis (ko00510)	0/0	1/60	9.8833e^-01^
78	Nicotinate and nicotinamide metabolism (ko00760)	0/0	1/40	6.4450e^-01^
79	Anthocyanin biosynthesis (ko00942)	0/0	1/1	7.1130e^-02^
80	Isoquinoline alkaloid biosynthesis (ko00950)	0/0	1/18	7.3558e^-01^
81	Aminoacyl-tRNA biosynthesis (ko00970)	0/0	1/48	9.7147e^-01^
82	Circadian rhythm- plant (ko04712)	0/0	1/25	8.4255e^-01^

^a^ number of differentially expressed genes (DEGs) enriched in a certain pathway

^b^number of the expressed genes enriched in the same pathway.

Protein synthesis is performed by ribosomes, which are composed of various proteins and long RNA chains, at sites where genetic information encoded in messenger RNA is translated into a polypeptide chain. Similar to the COG results that showed that the category of translation, ribosomal structure, and biogenesis occupied the highest number, the major pathways were also involved in the structure and composition of ribosomes (Ko03010), processing in the endoplasmic reticulum (Ko04141), and RNA transport (Ko03013).

The pathways involved in the synthesis and metabolism of carbohydrates also occupied a large part of DEGs such as glycolysis/gluconeogenesis (Ko00010), carbon fixation in photosynthetic organisms (Ko00710), and starch and sucrose metabolism (Ko00500). Additionally, the pathways of oxidative phosphorylation (Ko00190) and glycolysis/gluconeogenesis (Ko00010), which are involved in energy production, also showed enrichment.

Although only 3 and 4 DEGs were involved in plant hormone signal transduction (Ko04075) at both pollen mother cell formation and meiosis stages, 316 expressed unigenes were assigned to this pathway, indicating that plant hormones play a vital role in pollen abortion in Yu98-8A. Interestingly, four pathways, which included base excision repair (Ko03410), mismatch repair (Ko03430), nucleotide excision repair (Ko03420), and homologous recombination (Ko03440), showed enrichment during pollen mother cell formation stage compared to that in the meiosis stage, which showed no DEGs.

### Content analysis of starch and soluble sugar between MS and MF (WT) during anther development

In plants, as a non-photosynthetic organ, the anther obtains photosynthetic assimilates mainly from source organs to support pollen development and maturation through the phloem, which is responsible for transporting organic nutrients from source tissues to sink tissues such as roots and reproductive organs [22,23]. During the early stages of pollen development, large amounts of sugars are mobilized to anthers to support their development from source tissues [24]. Pollen development and maturation requires the accumulation of starch, which functions as an energy reserve for its use at relatively late stages such as that of germination [25]. Therefore, any disturbances in sugar synthesis, transportation, metabolism, and starch synthesis during pollen development could severely impair pollen development, resulting in male sterility [26].

**Fig 5:**
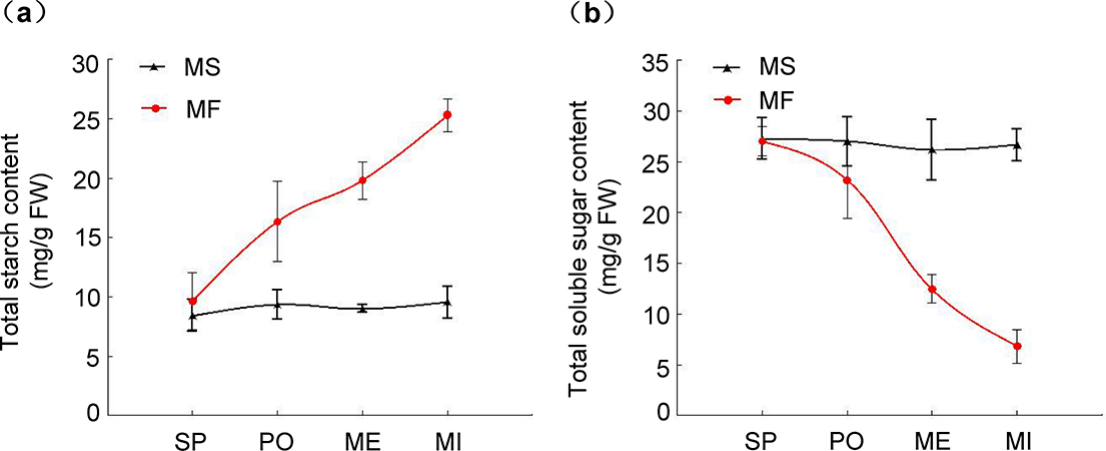
Content of total starch and soluble sugars in MF and MS buds. Data represent the mean and standard error from three replicates. (a) shows the content and profile of starch in buds during pollen development, and (b) shows that of soluble sugars in buds during the pollen development. SP, PO, ME, and MI represent the sporogenous cell, pollen mother cell formation, meiosis, and microsprore development stages, respectively. FW indicates fresh weight.

The present study monitored the dynamics of starch and soluble sugar content in the development stages of cotton plants. During the sporogenous cell stage, no siginicant differences in starch content between MS and MF buds were observed. However, from the pollen mother cell formation stage to the microspore development stage, the starch content of MS sustained almost the same low level of about 7 mg/g (FW) during whole pollen development which was lower than that of MF possibly due to its steady increase in MF buds (Fig 5a). No siginicant differences in soluble sugar content were observed between the MS and MF buds during the sporogenous cell stage. However, from the start of pollen mother cell formation stage, soluble sugars of MS buds kept in almost the same level compared to that of MF buds which drastically decreased relative to that of MS buds (Fig 5b). In contrast to that of MF buds, the content of both starch and soluble sugars were sustained (about 8 mg/g and 27 mg/g, respectively) during the whole developmental stages of sterile pollen. These results indicate the occurrence of certain disturbances in sugar metabolism and starch synthesis during pollen development of the genic MS line Yu98-8A.

**Fig 6:**
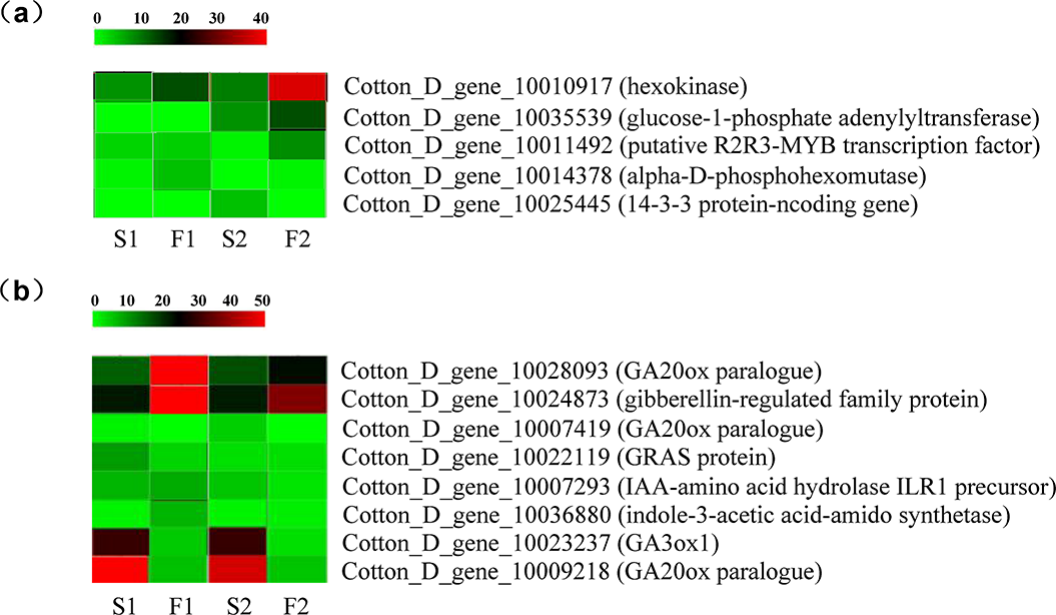
Detailed expression profile of DEGs involved in starch, sugars, and pytohormones. (a) and (b) represent the expression profiles of genes involved in soluble sugars/starch biogenesis and metabolism and phytohormones signaling, respectively. The relative expression profiles were obtained by RNA-seq after conducting equation and logarithmic transformations of reads per kilobase per million mapped reads (RPKM).

In agreement with the dynamics of starch and total soluble sugars and the phenotypic microscopic observations of MS and MF buds, several genes, including some housekeeping genes involved in sucrose and starch metabolism, were differentially expressed between the MS and MF buds. For instance, among the housekeeping genes, several genes, including hexokinase (ID: Cotton_D_gene_10010917), alpha-D-phosphohexomutase (ID: Cotton_D_gene_10014378), glucose-1-phosphate adenylyltransferase (ID: Cotton_D_gene_10035539), which are involved the metabolism of sucrose in starch biosynthesis, were significantly or extremely downregulated in MS buds at the meiosis stage [27,28]. Starch is synthesized in anthers prior to meiosis and is subsequently hydrolyzed to provide energy for lipid synthesis in both the tapetum and microspores [29]. Previous studies have indicated that 14-3-3 proteins were associated with ATP synthases in a phosphorylation-dependent style, playing a regulatory role in starch accumulation [30]. In maize and wolfberry, the downregulated expression of the14-3-3 protein leads to pollen sterility [31,32]. Accordingly, in the present study, the expression of 14-3-3 protein-coding gene (ID: Cotton_D_gene_10025445), which interactes with PM H^+^-ATPase to transport extracellular sugars into the developing pollen from the nutrient rich locular fluid, was upregulated during the pollen mother cell formation stage [33]. However, the expression of that gene was extensively downregulated at the meiosis stage compared to that in the MF buds. In addition, putative R2R3-MYB transcription factor (ID: Cotton_D_gene_10011492) (S4 Table) involved in regulating sugar partitioning during male reproductive development was upregulated at least two-fold during the meiosis stage in genic MS buds compared to normal development buds, indicating that process of sugar transport from source tissues to buds was not disrupted (Fig 6a). This may also be interpreted as having the same content of soluble sugars between MS and MF buds at the early stage of microsporogenesis. Therefore, although the transport of soluble sugars from nutrient-rich tissues to regions of microsporogenesis is normal at the early stage of pollen development in genic MS buds, the deficiency of starch due to the low expression of some genes involved in starch biosynthesis might be one of the main reasons for pollen sterility during the follow-up stages of pollen development in the genic MS line Yu98-8A.

### Dynamics of endogenous hormones during anther development

**Fig 7:**
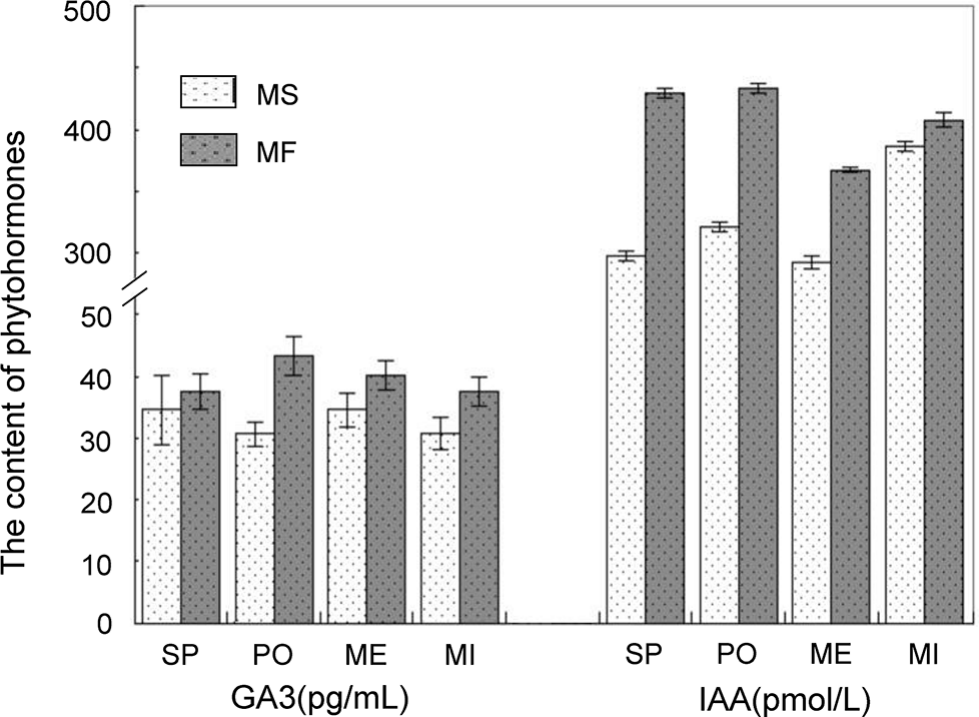
Content of different phytohormones in MF and MS buds. Data represent the mean and standard error from three replications. SP, PO, ME, and MI represent the sporogenous cell, pollen mother cell formation, meiosis and microspore development stages, respectively.

Plant hormones are important regulators of metabolism, and previous studies have shown that the occurrence of male sterility in plants is usually accompanied by changes in the levels of expression of endogenous hormones in various reproductive organs [34,35]. Accordingly, in the present study, the unigenes associated with plant hormones metabolism also showed a differentially expressed pattern during pollen mother cell formation and meiosis stages between MF and MS buds. The content of phytohormones, which include GA_3_ and indole-3-acetic acid (IAA), were measured by ELISA to monitor phytohormone dynamic changes during pollen development in MF and MS buds. The GA_3_ concentration of MS buds was lower than that of MF buds during the whole development period, and similarly, the IAA concentration of MS buds was also significantly lower than that of MF buds from the sporogenous cell stage to the meiosis stage (Fig 7).

Gibberellic acid (GAs) promote the development of flowers [36,37] and GA deficiency causes abnormal development of pollens, which usually leads to male sterility [38]. GA is an absolute requirement for flower initiation [39]. GA biosynthesis and accumulation is primarily dependent on the expression of *GA20ox* gene and *GA3ox1*, both of which are biosynthesis genes [40,41], and the expression of *GA2ox* gene, which inactivates the bioactive GAs and their precursors [42]. The expression pattern of these genes shows that, compared to the MF buds, although the *GA3ox1* paralogous gene (ID: Cotton_D_gene_10023237) was upregulated during the sporogenous cell stage in genic MS buds, the expression of the *GA20ox* paralogue (ID: Cotton_D_gene_10028093) was downregulated by about three-fold during the pollen mother cell formation stage in MS buds. In addition, the *GA2ox* paralogue (ID: Cotton_D_gene_10009218) was upregulated by at least more than 11-fold during the pollen mother cell formation stage in MS buds, and the another paralogue (ID: Cotton_D_gene_10007419) upregulated by at least 4.5-fold at the meiosis stage in MS buds. Gibberellin signaling promotes the growth and development of plants by degrading DELLA proteins, which are growth-suppressing members of the GRAS family. Accordingly, an analogue-encoding gene (ID: Cotton_D_gene_10022119) of that family was downregulated at the pollen meiosis stage in MS buds. In addition, the gibberellin-regulated family protein (ID: Cotton_D_gene_10024873) (S4 Table) was also significantly downregulated from the pollen mother cell formation to the meiosis stages because of the low GA content in genic MS buds.

Indole-3-acetic acid (IAA), the main natural form of auxin in plants, is also an essential plant hormone that plays a critical role in regulating many aspects of plant growth and development [43,44]. Previous reports have shown that the low content of IAA in abortive anthers is the main reason of male sterility [35]. In the present study, the content of IAA remained at a lower level during the whole pollen development period, especially from the sporogenous cell formation to meiosis stages (Fig 5). In addition, IAA can be modified by conjugation with sugars and amino acids, thus providing an effective means for controlling the pool of free hormones and simultaneously serving as the first step in the catabolic pathway [45,46]. Accordingly, in the present study, the RNA-seq data showed that the expression of an indole-3-acetic acid-amido synthetase gene (ID: Cotton_D_gene_10036880) in MS buds was downregulated by almost three-fold compared to that of MF buds during the meiosis stage. On the other hand, at the same development stage, the expression of another gene (ID: Cotton_D_gene_10007293) encoding an IAA-amino acid hydrolase ILR1 precursor, which releases active IAA from conjugates in MS buds, is significantly lower than that observed in MF buds (Fig 6b) [47,48]. Earlier research findings therefore suggest that IAA promotes GA biosynthesis at the final activation step, from *GA20* to *GA1* via *GA3ox* in pea, or the step from *GA19* to *GA20* via *GA20ox* in tobacco [49,50]. IAA derived from the developing panicle is necessary for the elongation of the uppermost internodes by promoting GA1 biosynthesis via the upregulation of the *OsGA3ox2* mRNA [51]. These observations suggest that IAA and GAs play distinct yet partially overlapping functions in regulating plant reproductive development.

## Conclusions

The application of RNA-seq in combination with microscopic observation has generated abundant and high-quality data for the biochemical and molecular analysis of early pollen abortion of a novel cotton MS mutant Yu98-8A. The main abortion period starts from pollen mother cell formation to the meiosis stage, and several genes associated with sugars and starch metabolism, oxidative phosphorylation, and plant endogenous hormone signaling are activated or suppressed before and during the meiosis stage in sterile buds. Further analysis has shown that phytohormones play a critical and complicated role in pollen abortion. However, the concrete molecular function of these genes remains elusive. The identification of DEGs involved in cotton pollen abortion can extend our understanding of the complex molecular events during this process, and provide a foundation for future researches on breeding for heterosis.

## Materials and Methods

### Plant materials

Plants of genic MS line Yu98-8A and the wild-type of the background with Yu98-8A were grown in the experimental field of the Henan Academy of Agricultural Science, Xinxiang, Henan province, China. The buds, without its bracts and measuring < 9 mm in diameter, were harvested from 10 sterile and 10 fertile plants at the same time to identify the correspondence between the diameter and different developmental stages using cytological methods. Buds within the specific diameter range (MS: 1.8–2.5 mm, MF: 2.0–2.8 mm) and (MS: 2.5–3.0 mm, MF: 2.8–3.3 mm) were again collected from at least 50 plants of each line. Some of these were snap-frozen in liquid nitrogen and stored at -80°C for future use, whereas the others were fixed in FAA [10% formalin, 5% acetic acid, 50% ethanol (v/v)] at room temperature for microscopic evaluation.

### Histological analysis

After the sterile and fertile floral buds were fixed, dehydration and infiltration of the specimen were performed using a series of ethanol gradations and paraffin. After embedding in paraffin, the samples were sliced into semi-thin (~8 μm) sections using a microtome. The sections were then stained with 0.1% toluidine blue O for 30–60 s at room temperature and were examined under a microscope.

### Measurement of starch and soluble sugar content

The floral buds were ground into a fine powder in solid nitrogen with a pestle and mortar. Bud powder (1 g) was dissolved in 20 μL of ddH_2_O in centrifuge tubes and incubated at 100°C for 10 min, then centrifuged at 2,500 g for 5 min. The supernatants and pellets were used in measuring the content of soluble sugars and starch as described elsewhere [52].

### Measurement of hormone levels

The extraction and purification of endogenous phytohormones prior to the immunoassay were conducted by enzyme-linked immunosorbent assay (ELISA) as described elsewhere with minor modification [53,54]. Briefly, 0.5 g of bud powder was homogenized through repeated inversion in pre-chilled 80% aqueous methanol containing butoylated hydroxytoluene (1 mmol/L) overnight at 4°C. The supernatants were centrifuged at 5,000 *g* for 20 min at 4°C, and the solid residue was re-extracted and re-centrifuged. The crude extracts were passed through a Sep-Pak C18 cartridge. Then, the filtrates were dried in N_2_ gas and the resultant residues were dissolved in phosphate buffered saline (PBS, 0.01 mol/L, pH 7.4). The levels of IAA and GA_3_ were then determined by using monoclonal antibodies. The absorbance of the developed color was measured at a wavelength of 490 nm using a microplate reader.

### RNA extraction, library construction, and RNA-seq

Total RNA was extracted from young floral buds using the pBiozol Total RNA Extraction Reagent according to the manufacturer’s protocol. The integrity of all the total RNAs was checked by 1% agarose gel electrophoresis and the concentration and purity were determined using a NanoDrop. The cDNA library preparation and sequencing reactions were conducted by the Biomarker Technology Company, Beijing, China. The RNA-Seq libraries were constructed using NEBNext mRNA Library Prep Master Mix Set for Illumina and NEBNext Multiplex Oligos for Illumina according to the manufacturer’s instructions.Suitable fragments, as judged by agarose 1.8% gel electrophoresis, were selected for use as templates for PCR amplification using Library Quantification Kit-Illumina GA Universal, cDNA libraries were sequenced on Illumina HiSeq2500 using paired-end technology.

### *De novo* assembly and DGE tags analysis

Sequencing output raw data were first filtered to remove adaptor tags, low quality sequences (tags with unknown sequences ‘N’) and tags with a copy number of 1 (probably sequencing error). The clean reads from each library were assembled separately using the Trinity method. Gene expression levels were measured using EBSeq software after numbers of reads and were normalized with RPKM [55,56] based on a false discovery rate (FDR) of < 0.01 and a fold change of ≥ 2 in RPKM between two libraries. Due to the use of a single biological replicate for each developmental stage, these high levels of significance may not reflect biological differences caused by development but may instead reflect other differences among the samples. Therefore, all the results of all statistical tests were corrected for multiple testing with the Benjamini-Hochberg with a P-value <0.001.

### Annotation and functional classification

To assign putative functions to assembled unigenes, a set of sequential BLAST searches against with sequences in NCBI (http://www.ncbi.nlm.nih.gov/protein, http://www.ncbi.nlm.nih.gov/nucleotide), Swiss-Prot (http://www.ebi.ac.uk/uniprot), GO (http://www.geneontology.org/), COG (http://www.ncbi.nlm.nih.gov/COG/), and KEGG (http://www.genome.jp/kegg/ko.html) databases were performed, respectively [57, 58]. In order to further elucidate distributions of DEGs based on their functions and belonged biological pathways, in addition to COG annotation, the KEGG enrichment analyses with FDR q-value ≤ 0.05 was performed [59].

### qRT-PCR validation

qRT-PCR was carried out to estimate the validity of RNA-Seq technology for expression profile analysis. Gene-specific primers were designed according to the cDNAs sequences with Primer Premier 5.0 (S3 Table). The qRT-PCR assay was performed in triplicate on an ABI Prism 7000 Real-time PCR system. The reactions were incubated in a 96-well plate at 95°C for 10 min, followed by 40 cycles of 95°C for 15 s and 60°C for 60 s. The cotton endogenous *Actin* gene (Forwad primer: 5'-GATTCCGGTGACGGTGTTTC-3'; Reverse primer: 5'- TTCATCAAGGCATCGGTTAG-3') was used to normalize the amount of gene-specific RT-PCR products, and the relative expression levels of genes were calculated by using the 2^−ΔΔCt^ method.

## Supporting Information

S1 Fig Sequencing saturation curve analysis (RPKM≥0.1). X-axis and Y-axis represent represents the sequenced reads (M) and the the numbers (K) of expressed genes in each library, respectively.
(TIF)

S2 Fig Comparison of differential expression patterns of unigenes between MS and MF buds. The green dots represent DEGs, the red dots represent no DEGs. MF1 and MS1 represent expressed unigenes at mother pollen cell formation stage, and MF2 and MS2 represent that at the meiosis stage. FDR and FC represent the false discovery rate and fold change of differentially expressed unigenes.
(TIF)

S1 File Data of unigene sequences assembled by *de novo* assembly. (XLSX)

S1 Table Statistics and evaluations of RNA-seq reads from four libraries. (DOC)

S2 Table Assembly of RNA-seq reads from four libraries. (DOC)

S3 Table List of selected DEGs and corresponding primers used for real time RT-PCR analysis. (DOC)

S4 Table ID list of unigenes used in this study and the corresponding assembled unigene numbers. (DOC)

## References

[1] Sunilkumar G, Campbell LM, Puckhaber L, Stipanovic RD, Rathore KS (2006) Engineering cottonseed for use in human nutrition by tissue-specific reduction of toxic gossypol. Proc Natl Acad Sci USA 103(48):18054–18059. 17110445 10.1073/pnas.0605389103 PMC1838705

[2] Zhang XQ, Wang XD, Zhu YG, Zhu W, Jiang PD (2007) Breeding for male-sterility line with G. barbadense nuclear background and cytological observation of its microsporogenesis. Agricultural Sciences in China 6(5): 529–535.

[3] Zhao HY, Huang JL (2012) Study on microspore abortion of male sterile cotton Yamian A and Yamian B. Scientia Agricultura Sinica 45(20): 4130–4140.

[4] Song XL, Sun XZ, Wang MM, Liu YX, Liu JH (2003) Preliminary study on the changes of POD activity and phytohormones in anthers of double recessive genetic sterile line of cotton (Gossypium hirsutum L.). Scientia Agricultura Sinica 36 (7): 861–863.

[5] Jiang PD, Zhang XQ, Zhu YG, Zhu W, Xie HY, Wang XD (2007) Metabolism of reactive oxygen species in cotton cytoplasmic male sterility and its restoration. Plant Cell Reports 26:1627–1634. 17426978 10.1007/s00299-007-0351-6

[6] Ma XD, Xing CZ, Guo LP, Gong YC, Wang HL, Zhao YL, et al (2007) Analysis of differentially expressed genes in genic male sterility cotton (Gossypium hirsutum L.) using cDNA-AFLP. Journal of Genetics and Genomics 34(6): 536–543. 17601613 10.1016/S1673-8527(07)60059-9

[7] Ma JH, Wei HL, Song MZ, Pang CY, Liu J, Wang L, et al (2012) Transcriptome profiling analysis reveals that flavonoid and ascorbate-glutathione cycle are important during anther development in Upland Cotton. PLoS One 7(11): e49244 10.1371/journal.pone.0049244 23155472 PMC3498337

[8] Yang P, Han JF, Huang JL (2014) Transcriptome sequencing and de novo analysis of cytoplasmic male sterility and maintenance in JA-CMS cotton. PLoS ONE 9(11): e112320 10.1371/journal.pone.0112320 25372034 PMC4221291

[9] Sheng TZ (1989) Thesises on male sterile of cotton Chengdu: Sichuan Science and Technology Press.

[10] Yang XJ, Xie DY, Zhao YM, Li W, Zhao FA, Duan ZZ, et al (2013) Breeding and identification of herbicide-resistant genic male sterile line Yu98-8A1 of cotton (Gossypium hirsutum L.). Journal of Plant Genetic Resources 14(4):723–727.

[11] Zhao YM, Yang XJ, Fang WP, Xie DY, Yu YB, Zhao FA, et al (2012) Genetic analysis of new Genic male-sterile cotton line Yu 98-8A. Chinese Agricultural Science Bulletin 28(33): 98–102.

[12] Wang Z, Gerstein M, Snyder M (2009) RNA-Seq: a revolutionary tool for transcriptomics. Nat Rev Genet 10: 57–63. 10.1038/nrg2484 19015660 PMC2949280

[13] Yan XH, Dong CH, Yu JY, Liu WH, Jiang CH, Liu J, et al (2013) Transcriptome profile analysis of young floral buds of fertile and sterile plants from the self-pollinated offspring of the hybrid between novel restorer line NR1 and Nsa CMS line in Brassica napus. BMC Genomics 14: 26 10.1186/1471-2164-14-26 23324545 PMC3556089

[14] Liu C, Ma N, Wang PY, Fu N, Shen HL (2013) Transcriptome sequencing and De Novo analysis of a cytoplasmic male sterile line and its near-isogenic restorer line in chili pepper (Capsicum annuum L.). PLoS One 8: e65209 10.1371/journal.pone.0065209 23750245 PMC3672106

[15] Wei MM, Song MZ, Fan SL, Yu SX (2013) Transcriptomic analysis of differentially expressed genes during anther development in genetic male sterile and wild type cotton by digital gene-expression profiling. Genomics 14:97 10.1186/1471-2164-14-97 23402279 PMC3599889

[16] Grabherr MG, Haas BJ, Yassour M, Levin JZ, Thompson DA, Amit I, et al (2011) Full-length transcriptome assembly from RNA-Seq data without a reference genome. Nature Biotechnology (29): 644–652. 10.1038/nbt.1883 21572440 PMC3571712

[17] Langmead B, Trapnell C, Pop M, Salzberg SL (2009) Ultrafast and memory-efficient alignment of short DNA sequences to the human genome. Genome Biol 10: R25 10.1186/gb-2009-10-3-r25 19261174 PMC2690996

[18] Tatusov RL, Galperin MY, Natale DA, Koonin EV (2000) The COG database: a tool for genome-scale analysis of protein functions and evolution. Nucleic Acids Res 28(1): 33–36. 10592175 10.1093/nar/28.1.33 PMC102395

[19] Kanehisa M, Goto S, Kawashima S, Okuno Y, Hattori M (2004) The KEGG resource for deciphering the genome. Nucleic Acids Res 32: D277–D280. 14681412 10.1093/nar/gkh063 PMC308797

[20] Altschul SF, Madden TL, Schäffer AA, Zhang J, Zhang Z, Miller W, et al (1997) Gapped BLAST and PSI-BLAST: A new generation of protein database search programs. Nucleic Acids Res 25(17): 3389 9254694 10.1093/nar/25.17.3389 PMC146917

[21] Wang DX, Oses-Prieto JA, Li KH, Fernandes JF, Burlingame AL, Walbot V (2010) The male sterile 8 mutation of maize disrupts the temporal progression of the transcriptome and results in the mis-regulation of metabolic functions. The Plant Journal 63: 939–951. 10.1111/j.1365-313X.2010.04294.x 20626649 PMC2974755

[22] Goetz M, Godt DE, Guivarc’h A, Kahmann U, Chriqui D, Roitsch T (2001) Induction of male sterility in plants by metabolic engineering of the carbohydrate supply. Proc Natl Acad Sci USA 98: 6522–6527. 11371651 10.1073/pnas.091097998 PMC33501

[23] Zhang H, Liang WQ, Yang XJ, Luo X, Jiang Ning, Ma Hong, et al (2010) Carbon starved anther encodes a MYB domain protein that regulates sugar partitioning required for rice pollen development. Plant Cell 22: 672–689. 10.1105/tpc.109.073668 20305120 PMC2861464

[24] Oliver SN, Van Dongen JT, Alfred SC, Mamun EA, Zhao X, Saini HS, et al (2005) Coldinduced repression of the rice anther-specific cell wall invertase gene OSINV4 is correlated with sucrose accumulation and pollen sterility. Plant Cell Environ 28: 1534–1551.

[25] Datta R, Chamusco KC, Chourey PS (2002) Starch biosynthesis during pollen maturation is associated with altered patterns of gene expression in maize. Plant Physiol 130: 1645–1656. 12481048 10.1104/pp.006908 PMC166680

[26] Mamun EA, Alfred S, Cantrill LC, Overall RL, Sutton BG (2006) Effects of chilling on male gametophyte development in rice. Cell Biol Int 30: 583–591. 16730464 10.1016/j.cellbi.2006.03.004

[27] Tsai CY, Salamini F, Nelson OE (1970) Enzymes of carbohydrate metabolism in the developing endosperm of maize. Plant Physiol 46: 299–306. 16657454 10.1104/pp.46.2.299 PMC396583

[28] Nelson O, Pan D (1995) Starch synthesis in maize endosperm. Annu Rev Plant Physiol Plant Mol Biol 46: 475–496.

[29] Vizcay-Barrena G, Wilson ZA (2006) Altered tapetal PCD and pollen wall development in the Arabidopsis ms1 mutant. J Exp Bot 57: 2709–2717. 16908508 10.1093/jxb/erl032

[30] Sehnke PC, Chung HJ, Wu K, Ferl RJ (2001) Regulation of starch accumulation by granule-associated plant 14-3-3 proteins. Proc Natl Acad Sci USA 98: 765–770. 11149942 10.1073/pnas.021304198 PMC14662

[31] Datta R, Chamusco KC, Chourey PS (2002) Starch biosynthesis during pollen maturation is associated with altered patterns of gene expression in maize. Plant Physiol 130: 1645–1656. 12481048 10.1104/pp.006908 PMC166680

[32] Zheng R, Yue SJ, Xu XY, Liu JY, Xu Q, Wang XL, et al (2012) Proteome analysis of the wild and YX-1 male sterile mutant anthers of wolfberry (Lycium barbarum L.). PLoS One 7(7): e41861 10.1371/journal.pone.0041861 22860020 PMC3408462

[33] Jahn T, Fuglsang AT, Olsson A, Bruntrup IM, Collinge DB, Volkmann D, et al (1997) The 14-3-3 protein interacts directly with the C-terminal region of the plant plasma membrane H^+^-ATPase. Plant Cell 9: 1805–1814. 9368417 10.1105/tpc.9.10.1805 PMC157023

[34] Shukla A, Sawhney V K (1994) Abscisic acid: one of the factors affecting male sterility in Brassi canapus. Physiologia Plantarum 91: 522–528.

[35] Singh S, Sawhney VK (1992) Endogenous hormones in seeds, germination behaviour early seedling characteritics in a normal an ogura cytoplasmid male sterile line of rapeseed (Brassica napus L.). J Exp Bot 43: 1497–1505.

[36] Bagnall DJ (1992) Control of flowering in Arabidopsis thaliana by light, vernalisation and gibberellins. Plant Physiol 19: 401–409.

[37] Pharis RP, King RW (1985) Gibberellins and reproductive development in seed plants. Annu Rev Plant Physiol 36: 517–568.

[38] Andersen JR, Schrag T, Melchinger AE, Zein I, Lübberstedt T (2005) Validation of Dwarf 8 polymorphisms associated with flowering time in elite European inbred lines of maize (Zea mays L.). Theor Appl Genet 111(2): 206–17. 15933874 10.1007/s00122-005-1996-6

[39] King RW, Moritz T, Evans LT, Martin J, Andersen CH, Blundell C, et al (2006) Regulation of flowering in the long-day grass Lolium temulentum by gibberellins and the FLOWERING LOCUS T gene. Plant Physiol 141, 498–507. 16581877 10.1104/pp.106.076760 PMC1475477

[40] Lee DJ, Zeevaart JAD (2007) Regulation of gibberellin 20-oxidase1 expression in spinach by photo-period. Planta 226: 35–44. 17216482 10.1007/s00425-006-0463-1

[41] Sakata T, Oda S, Tsunaga Y, Shomura H, Kawagishi-Kobayashi M, Aya K, et al (2014) Reduction of gibberellin by low temperature disrupts pollen development in rice. Plant Physiol 164: 2011–2019. 10.1104/pp.113.234401 24569847 PMC3982758

[42] Sakamoto T, Kobayashi M, Itoh H, Tagiri A, Kayano T, Tanaka H, et al (2001) Expression of a gibberellin 2-oxidase gene around the shoot apex is related to phase transition in rice. Plant Physiol 125: 1508–1516. 11244129 10.1104/pp.125.3.1508 PMC65628

[43] Pagnussat GC, Alandete-Saez M, Bowman JL, Sundaresan V (2009) Auxin-dependent patterning and gamete specification in the Arabidopsis female gametophyte. Science 324: 1684–1689. 10.1126/science.1167324 19498110

[44] Zhao Y. (2010) Auxin biosynthesis and its role in plant development. Annu. Rev. Plant Biol. 61, 49–64. 10.1146/annurev-arplant-042809-112308 20192736 PMC3070418

[45] Normanly J, Slovin JP, Cohen JD (1995) Rethinking auxin biosynthesis and metabolism. Plant Physiol 107: 323–329. 12228361 10.1104/pp.107.2.323 PMC157132

[46] Normanly J (2010) Approaching cellular and molecular resolution of auxin biosynthesis and metabolism. Cold Spring Harb Perspect Biol 2: a001594 10.1101/cshperspect.a001594 20182605 PMC2827909

[47] Bartel B, Fink GR (1995) ILR1, an amidohydrolase that releases active indole-3-acetic acid from conjugates. Science 268:1745–1748. 7792599 10.1126/science.7792599

[48] LeClere S, Tellez R, Rampey RA, Matsuda SPT, Bartel B (2002) Characterization of a family of IAA-amino acid conjugate hydrolases from Arabidopsis. J Biol Chem 277: 20446–20452. 11923288 10.1074/jbc.M111955200

[49] Ross JJ, O’Neill DP, Wolbang CM, Symons GM, Reid JB (2002) Auxin-gibberellin interactions and their role in plant growth. Journal of Plant Growth Regulation 20: 346–353. 10.1007/s003440010034 11986760

[50] Wolbang CM, Chandler PM, Smith JJ, Ross JJ (2004) Auxin from the developing inflorescence is required for the biosynthesis of active gibberellins in barley stems. Plant Physiol 134: 769–776. 14730077 10.1104/pp.103.030460 PMC344552

[51] Yin CX, Gan LJ, Ng D, Zhou X, Xia K (2007) Decreased panicle-derived indole-3-acetic acid reduces gibberellin A1 level in the uppermost internode, causing panicle enclosure in male sterile rice Zhenshan 97A. J Exp Bot 58(10): 2441–2449. 17556768 10.1093/jxb/erm077

[52] Fu ZD, Zhang ZL, Qu WQ (2004) Metabolism: Experiments of plant physiology. Higher Education Harbor Higher Education Press.

[53] Yang J, Zhang J, Wang Z, Zhu Q, Wang W (2001) Hormonal changes in the grains of rice subjected to water stress during grain filling. Plant Physiol 127: 315–323. 11553759 10.1104/pp.127.1.315 PMC117987

[54] Cui D, Neill SJ, Tang Z, Cai W (2005) Gibberellin-regulated XET is differentially induced by auxin in rice leaf sheath bases during gravitropic bending. J Exp Bot 56: 1327–1334. 15767322 10.1093/jxb/eri133

[55] Leng N, Dawson JA, Thomson JA, Ruotti V, Rissman AI, Smits BM, et al (2013) EBSeq: an empirical Bayes hierarchical model for inference in RNA-seq experiments. Bioinformatics 29: 1035–1043. 10.1093/bioinformatics/btt087 23428641 PMC3624807

[56] Mortazavi A, Williams BA, McCue K, Schaeffer L, Wold B (2008) Mapping and quantifying mammalian transcriptomes by RNA-Seq. Nat Methods 5: 621–628. 10.1038/nmeth.1226 18516045 PMC13303166

[57] Altschul SF, Madden TL, Schäffer AA, Zhang J, Zhang Z, Miller W, et al (1997) Gapped BLAST and PSI-BLAST: a new generation of protein database search programs. Nucleic Acids Res 25: 3389–3402. 9254694 10.1093/nar/25.17.3389 PMC146917

[58] Conesa A, Götz S, García-Gómez JM, Terol J, Talón M, Robles M (2005) Blast2GO: a universal tool for annotation, visualization and analysis in functional genomics research. Bioinformatics 21: 3674–3676. 16081474 10.1093/bioinformatics/bti610

[59] Xie C, Mao X, Huang J, Ding Y, Wu J, Dong S, et al (2011) KOBAS 2.0: a web server for annotation and identification of enriched pathways and diseases. Nucleic Acids Res 39: W316–322. 10.1093/nar/gkr483 21715386 PMC3125809

