# Supplementary figures and images for: Transcriptomic Profiling Reveals Complex Molecular Regulation in Cotton Genic Male Sterile Mutant Yu98-8A
Source: PLoS One. 2015 Sep 18;10(9):e0133425. doi: 10.1371/journal.pone.0133425 (PMC4575049; doi:10.1371/journal.pone.0133425)

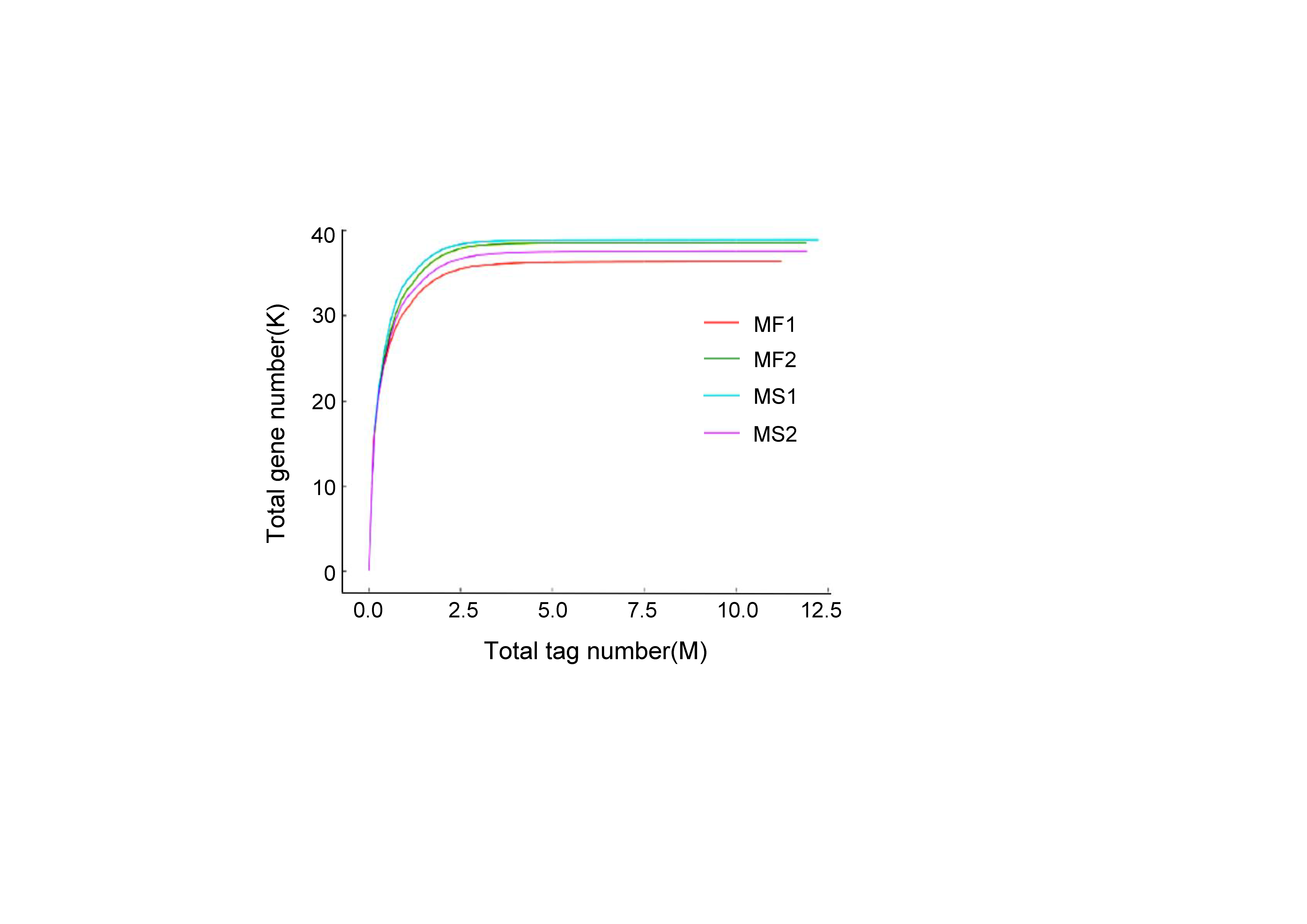

Supplement: S1 Fig — X-axis and Y-axis represent represents the sequenced reads (M) and the the numbers (K) of expressed genes in each library, respectively. (TIF) [file pone.0133425.s001.tif]

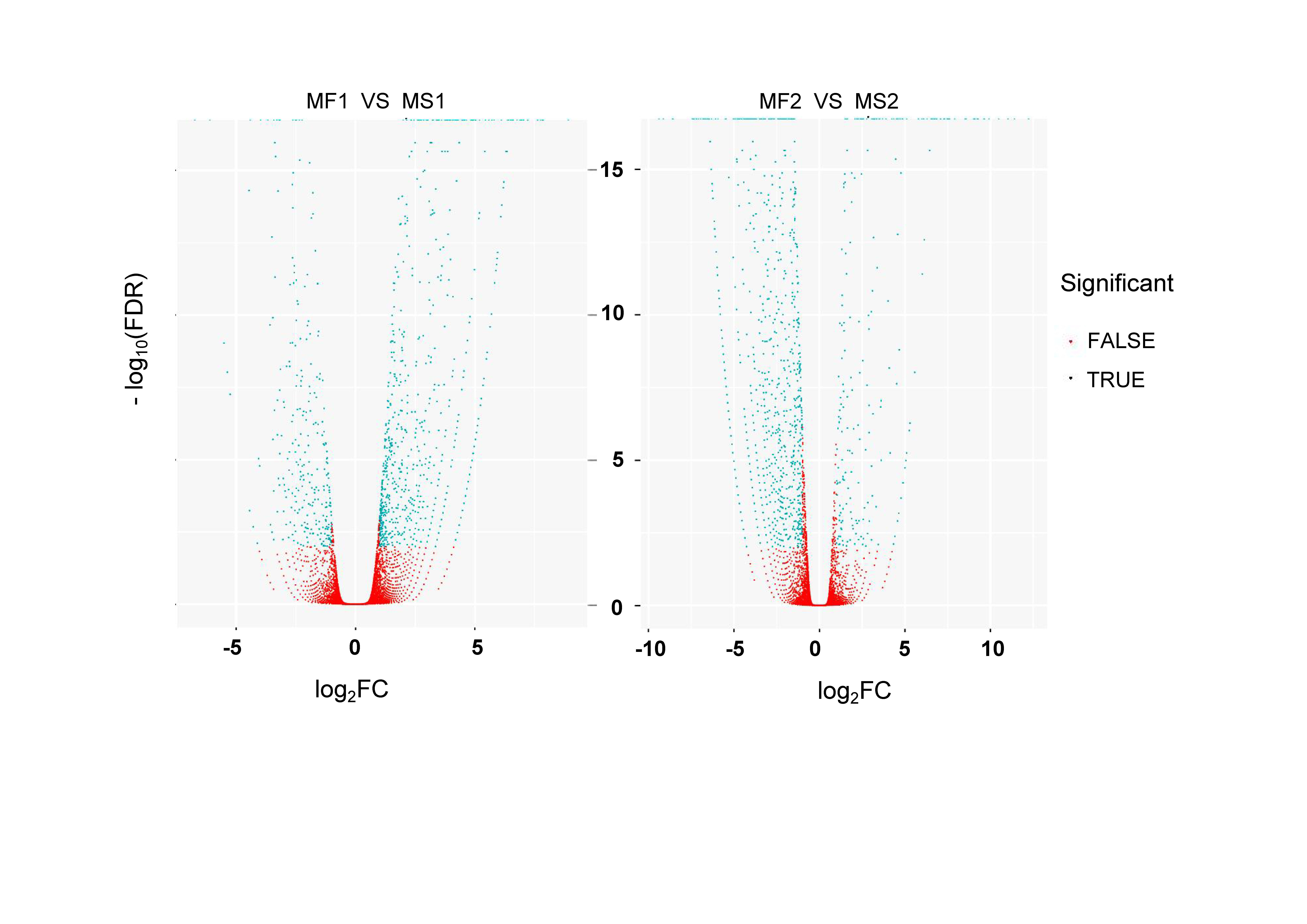

Supplement: S2 Fig — The green dots represent DEGs, the red dots represent no DEGs. MF1 and MS1 represent expressed unigenes at mother pollen cell formation stage, and MF2 and MS2 represent that at the meiosis stage. FDR and FC represent the false discovery rate and fold change of differentially expressed unigenes. (TIF) [file pone.0133425.s002.tif]
