# Supplementary material for: Transcriptomic Profiling Reveals Complex Molecular Regulation in Cotton Genic Male Sterile Mutant Yu98-8A
Source: PLoS One. 2015 Sep 18;10(9):e0133425. doi: 10.1371/journal.pone.0133425 (PMC4575049; doi:10.1371/journal.pone.0133425)
Supplement: S1 Table — (DOC) [file pone.0133425.s004.doc]

**S1 Table. Statistics and evaluations of RNA-seq reads from four libraries.**

| **Length range** | **Total reads** | **Total bases (nt)** | **GC%** | **Q30%** | **Total mapping reads** |
| --- | --- | --- | --- | --- | --- |
| MF1 | 11,241,020 | 2,270,488,050 | 45.20% | 86.55% | 8,987,878 (79.96%) |
| MF2 | 11,922,046 | 2,407,804,320 | 45.30% | 86.24% | 9,362,791 (78.53%) |
| MS1 | 12,247,449 | 2,473,784,586 | 44.49% | 86.19% | 9,355,748 (76.39%) |
| MS2 | 11,942,988 | 2,412,298,410 | 44.51% | 85.55% | 9,368,137 (78.44%) |

^a^the percentage of both base G and base C of all bases in sequencing results, ^b^the percentage of bases of high Q-value (≥30) in all the bases with any Q-values, ^c^the number of reads aligned to unigenes database, and the bracket is the percentage of the aligned reads in all the sequencing reads.
