# Supplementary material for: Transcriptomic Profiling Reveals Complex Molecular Regulation in Cotton Genic Male Sterile Mutant Yu98-8A
Source: PLoS One. 2015 Sep 18;10(9):e0133425. doi: 10.1371/journal.pone.0133425 (PMC4575049; doi:10.1371/journal.pone.0133425)
Supplement: S2 Table — (DOC) [file pone.0133425.s005.doc]

**S2 Table. Assembly of RNA-seq reads from four libraries.**

| **Length range** | **Contigs** | **Transcripts** | **Unigenes** |
| --- | --- | --- | --- |
| 200-300 | 1,895,538 (97.70%) | 16,974 (17.27%) | 12,223 (27.28%) |
| 300-500 | 17,756 (0.92%) | 18,152 (18.46%) | 10,403 (23.22%) |
| 500-1,000 | 13,670 (0.70%) | 24,200 (24.62%) | 9,463 (21.12%) |
| 1,000-2,000 | 9,092 (0.47%) | 25,461 (25.90%) | 8,292 (18.51%) |
| 2,000+ | 4,070 (0.21%) | 13,518 (13.75%) | 4,423 (9.87%) |
| Total number | 1,940,126 | 98,305 | 44,804 |
| Total length | 137,659,428 | 103,657,641 | 38,392,861 |
| N50 length | 82 | 1,609 | 1,456 |
| Mean length | 70.95 | 1,054.45 | 856.91 |
