# Supplementary material for: Transcriptomic Profiling Reveals Complex Molecular Regulation in Cotton Genic Male Sterile Mutant Yu98-8A
Source: PLoS One. 2015 Sep 18;10(9):e0133425. doi: 10.1371/journal.pone.0133425 (PMC4575049; doi:10.1371/journal.pone.0133425)
Supplement: S3 Table — (DOC) [file pone.0133425.s006.doc]

**S3 Table. List of selected DEGs and corresponding primers used for real time RT-PCR analysis.**

| **No** | **Gene ID (Gene name or molecular function)** | **Forward primer(5' → 3')** | **Reverse primer(5'→ 3')** |
| --- | --- | --- | --- |
| 1 | [Cotton_D_gene_10025846](http://cgp.genomics.org.cn/page/cn/mapview.jsp?dbKey=cotton_d&refId=4717&start=1505792&end=1506100) (Calcium-binding protein) | TGCTCCAAAGTGTTGTGAAA | GGACCCTAGAAGTGGAATGTA |
| 2 | [Cotton_D_gene_10008857](http://cgp.genomics.org.cn/page/cn/mapview.jsp?dbKey=cotton_d&refId=4718&start=19989445&end=19989723) (Pollen-specific protein) | GCCGAATCCTTACTTCCTTG | GTTGCGTTAATATCCACCCT |
| 3 | [Cotton_D_gene_10009218](http://cgp.genomics.org.cn/page/cn/mapview.jsp?dbKey=cotton_d&refId=6417&start=607248&end=609632) (GA dioxygenase) | AAAAGGCAGCAAGCAAGGAA | GGGATAAGCGGAGGGAACAA |
| 4 | [Cotton_D_gene_10024447](http://cgp.genomics.org.cn/page/cn/genedetail.jsp?id=65423&dbKey=cotton_d) (ABA hydroxylase) | ATGATGCTGCTTTGGCGTTAG | TCATTGAGGGCATTGAGGTG |
| 5 | [Cotton_D_gene_10012996](http://cgp.genomics.org.cn/page/cn/mapview.jsp?dbKey=cotton_d&refId=4721&start=26846488&end=26846904) (Cyclin kinase inhibitor) | AGTAAGCCCTAAGCAACTCCA | CATCACCATCATATCTTCTCCC |
| 6 | [Cotton_D_gene_10032583](http://cgp.genomics.org.cn/page/cn/mapview.jsp?dbKey=cotton_d&refId=7009&start=3201689&end=3202357) (ET transcription factor) | CTTCCTAACTCGCTTATCCTCC | CCCTGTTCGCCTTCCTCTTC |
| 7 | [Cotton_D_gene_10023237](http://cgp.genomics.org.cn/page/cn/mapview.jsp?dbKey=cotton_d&refId=4721&start=1824880&end=1826415) (GA dioxygenase) | ATGATGCTGCTTTGGCGTTAG | TCATTGAGGGCATTGAGGTG |
| 8 | [Cotton_D_gene_10016475](http://cgp.genomics.org.cn/page/cn/mapview.jsp?dbKey=cotton_d&refId=4717&start=4675012&end=4678684) (ABA hydroxylase) | AGGCGGCTAGGTTCGTGTT | AGTGCGTGAGGACCGATGA |
| 9 | [Cotton_D_gene_10025900](http://cgp.genomics.org.cn/page/cn/mapview.jsp?dbKey=cotton_d&refId=4717&start=2003729&end=2004362) (GA transcript protein) | CGGTGAGGTGCTCAAAGG | ATGCCCAAATGTCCCAGA |
| 10 | [Cotton_D_gene_10040607](http://cgp.genomics.org.cn/page/cn/mapview.jsp?dbKey=cotton_d&refId=4718&start=51442485&end=51443195) (Auxin responsive SAUR protein) | AAGCGAGGCTATCCAAAGAT | GGTGATTTCCTCCCCAACTA |
| 11 | [Cotton_D_gene_10025875](http://cgp.genomics.org.cn/page/cn/mapview.jsp?dbKey=cotton_d&refId=4717&start=1789828&end=1790298) (ET transcription factor) | CGGACGGACCTCGTAAGTAG | GGTGGCTGAAATAAGGGAAC |
| 12 | [Cotton_D_gene_10024873](http://cgp.genomics.org.cn/page/cn/mapview.jsp?dbKey=cotton_d&refId=4722&start=9431600&end=9433151) (Gibberellin-regulated) | TGTTGTAGCAAGGGCAGAC | CAAGAAGCCATGCCTATTC |
| 13 | [Cotton_D_gene_10009412](http://cgp.genomics.org.cn/page/cn/mapview.jsp?dbKey=cotton_d&refId=8982&start=609309&end=610041) (Pollen-specific protein) | TTATGCAAAGCGTAGAACACC | CCTCAGCTACACCACCCTCAC |
| 14 | Cotton_D_gene_10006155 (Pollen allergen protein) | ACAAGGGCTACCAATACCAAT | ATGTGACTGAAGCGCCATCTA |
| 15 | [Cotton_D_gene_10006550](http://cgp.genomics.org.cn/page/cn/mapview.jsp?dbKey=cotton_d&refId=4725&start=24105735&end=24108800) (asparagine synthetase) | CCAAACCGAACCATCAAGTC | GCTGGATACCCGTGACAACA |
| 16 | [Cotton_D_gene_10027147](http://cgp.genomics.org.cn/page/cn/mapview.jsp?dbKey=cotton_d&refId=4718&start=32052724&end=32053622) (IAA-induced protein) | ACCCGAGAACTGTCCCAATG | GCAAGCAACCAGGTCACCCT |
