# Supplementary material for: Transcriptomic Profiling Reveals Complex Molecular Regulation in Cotton Genic Male Sterile Mutant Yu98-8A
Source: PLoS One. 2015 Sep 18;10(9):e0133425. doi: 10.1371/journal.pone.0133425 (PMC4575049; doi:10.1371/journal.pone.0133425)
Supplement: S4 Table — (DOC) [file pone.0133425.s007.doc]

**S4 Table. ID list and annotation of unigenes used in this study and the corresponding assembled unigene numbers.**

| No | **Gene ID**^a^ | **Gene annotation**^b^ | **Unigene coding by RNA-seq**^c^ |
| --- | --- | --- | --- |
| 1 | [Cotton_D_gene_10025846](http://cgp.genomics.org.cn/page/cn/mapview.jsp?dbKey=cotton_d&refId=4717&start=1505792&end=1506100) | Calcium-binding protein | comp44399_c0 |
| 2 | [Cotton_D_gene_10008857](http://cgp.genomics.org.cn/page/cn/mapview.jsp?dbKey=cotton_d&refId=4718&start=19989445&end=19989723) | Pollen-specific protein | comp21716_c0 |
| 3 | [Cotton_D_gene_10009218](http://cgp.genomics.org.cn/page/cn/mapview.jsp?dbKey=cotton_d&refId=6417&start=607248&end=609632) | GA dioxygenase | comp47810_c1 |
| 4 | [Cotton_D_gene_10024447](http://cgp.genomics.org.cn/page/cn/genedetail.jsp?id=65423&dbKey=cotton_d) | ABA hydroxylase | comp40780_c0 |
| 5 | [Cotton_D_gene_10012996](http://cgp.genomics.org.cn/page/cn/mapview.jsp?dbKey=cotton_d&refId=4721&start=26846488&end=26846904) | Cyclin kinase inhibitor | comp46627_c0 |
| 6 | [Cotton_D_gene_10032583](http://cgp.genomics.org.cn/page/cn/mapview.jsp?dbKey=cotton_d&refId=7009&start=3201689&end=3202357) | ET transcription factor | comp43151_c0 |
| 7 | [Cotton_D_gene_10023237](http://cgp.genomics.org.cn/page/cn/mapview.jsp?dbKey=cotton_d&refId=4721&start=1824880&end=1826415) | GA dioxygenase | comp25251_c0 |
| 8 | [Cotton_D_gene_10016475](http://cgp.genomics.org.cn/page/cn/mapview.jsp?dbKey=cotton_d&refId=4717&start=4675012&end=4678684) | ABA hydroxylase | comp45968_c0 |
| 9 | [Cotton_D_gene_10025900](http://cgp.genomics.org.cn/page/cn/mapview.jsp?dbKey=cotton_d&refId=4717&start=2003729&end=2004362) | GA transcript protein | comp42436_c0 |
| 10 | [Cotton_D_gene_10040607](http://cgp.genomics.org.cn/page/cn/mapview.jsp?dbKey=cotton_d&refId=4718&start=51442485&end=51443195) | Auxin responsive SAUR protein | comp40120_c1 |
| 11 | [Cotton_D_gene_10025875](http://cgp.genomics.org.cn/page/cn/mapview.jsp?dbKey=cotton_d&refId=4717&start=1789828&end=1790298) | ET transcription factor | comp43241_c0 |
| 12 | [Cotton_D_gene_10024873](http://cgp.genomics.org.cn/page/cn/mapview.jsp?dbKey=cotton_d&refId=4722&start=9431600&end=9433151) | Gibberellin-regulated | comp42152_c0 |
| 13 | [Cotton_D_gene_10009412](http://cgp.genomics.org.cn/page/cn/mapview.jsp?dbKey=cotton_d&refId=8982&start=609309&end=610041) | Pollen-specific protein | comp45707_c0 |
| 14 | Cotton_D_gene_10006155 | Pollen allergen protein | comp45409_c0 |
| 15 | [Cotton_D_gene_10006550](http://cgp.genomics.org.cn/page/cn/mapview.jsp?dbKey=cotton_d&refId=4725&start=24105735&end=24108800) | Asparagine synthetase | comp38864_c0 |
| 16 | [Cotton_D_gene_10027147](http://cgp.genomics.org.cn/page/cn/mapview.jsp?dbKey=cotton_d&refId=4718&start=32052724&end=32053622) | IAA-induced protein | comp42783_c0 |
| 17 | [Cotton_D_gene_10014378](http://cgp.genomics.org.cn/page/cn/mapview.jsp?dbKey=cotton_d&refId=4724&start=18864894&end=18867963) | Alpha-D-phosphohexomutase | comp30200_c0 |
| 18 | [Cotton_D_gene_10035539](http://cgp.genomics.org.cn/page/cn/mapview.jsp?dbKey=cotton_d&refId=4719&start=42367145&end=42370748) | Glucose-1-phosphate adenylyl transferase | comp48734_c0 |
| 19 | [Cotton_D_gene_10010917](http://cgp.genomics.org.cn/page/cn/mapview.jsp?dbKey=cotton_d&refId=5871&start=363256&end=367987) | Hexokinase | comp52314_c0 |
| 20 | [Cotton_D_gene_10025445](http://cgp.genomics.org.cn/page/cn/mapview.jsp?dbKey=cotton_d&refId=4726&start=2202862&end=2205398) | 14-3-3 protein | comp28811_c0 |
| 21 | [Cotton_D_gene_10011492](http://cgp.genomics.org.cn/page/cn/mapview.jsp?dbKey=cotton_d&refId=4728&start=7349902&end=7350726) | Putative R2R3-MYB transcription factor | comp45952_c0 |
| 22 | [Cotton_D_gene_10028093](http://cgp.genomics.org.cn/page/cn/mapview.jsp?dbKey=cotton_d&refId=4721&start=13597674&end=13599375) | GA20ox paralogue | comp53409_c0 |
| 23 | [Cotton_D_gene_10007419](http://cgp.genomics.org.cn/page/cn/mapview.jsp?dbKey=cotton_d&refId=4718&start=61946783&end=61951876) | GA2ox paralogue | comp100028_c0 |
| 24 | [Cotton_D_gene_10022119](http://cgp.genomics.org.cn/page/cn/mapview.jsp?dbKey=cotton_d&refId=4718&start=35804326&end=35805702) | DELLA protein | comp47394_c1 |
| 25 | [Cotton_D_gene_10036880](http://cgp.genomics.org.cn/page/cn/mapview.jsp?dbKey=cotton_d&refId=4719&start=29065986&end=29067981) | Indole-3-acetic acid-amido synthetase | comp49929_c1 |
| 26 | [Cotton_D_gene_10007293](http://cgp.genomics.org.cn/page/cn/mapview.jsp?dbKey=cotton_d&refId=4717&start=3908287&end=3911838) | Indole-3-acetic acid-amido synthetase | comp50257_c0 |

^a,b^ the homologous gene ID and the functional annotation of assembled unigene, ^c^ the number was named by authors and their corresponding sequences were stored in S1 File.
